# Increased Smad3 and reduced Smad2 levels mediate the functional switch of TGF-β from growth suppressor to growth and metastasis promoter through TMEPAI/PMEPA1 in triple negative breast cancer

**DOI:** 10.18632/genesandcancer.194

**Published:** 2019

**Authors:** Prajjal K. Singha, Srilakshmi Pandeswara, Hui Geng, Rongpei Lan, Manjeri A. Venkatachalam, Albert Dobi, Shiv Srivastava, Pothana Saikumar

**Affiliations:** ^1^ Department of Med/Hematology & Med Oncology, UT Health Science Center at San Antonio, TX, USA; ^2^ Department of Pathology, Center for Prostate Disease Research, Department of Surgery, Uniformed Services University of the Health Sciences, Bethesda, MD, USA

**Keywords:** TGF-β, Smad2, Smad3, PTEN, TMEPAI

## Abstract

Screening of several TNBC cell lines showed altered Smad2 and Smad3 protein levels compared to normal mammary epithelial cells, suggesting the possibility that it could play an important role in the escape of cancer cells from TGF-β mediated growth inhibition. To assess the functional relevance of these endogenous molecules, Smad2 or Smad3 expression was knocked down individually and assessed their effects on pro-oncogenic properties of TGF-β. Smad3 deficiency reduced growth and invasion capacity of breast cancer cells in comparison to Smad2 which had no effect. Smad3 deficiency was also found to be associated with a reduction in the expressions of TMEPAI/PMEPA1 and EMT inducing transcription factors, E-Cadherin and increased expression of cell cycle inhibitors and Vimentin. On the other hand, Smad2 deficiency had opposite effect on these regulators. Interestingly, the decreased growth, invasion and associated gene expressions were largely reversed by overexpressing TMEPAI in Smad3 knockdown cells, suggesting that Smad3-TMEPAI axis may be involved in subverting growth suppressive effects of TGF-β into growth promotion. Similarly, altered levels of Smad proteins and TMEPAI were also noted in primary TNBC tumor tissues. Analysis of the existing databases provided additional support in terms of TMEPAI and Smad2 expression impacting the survival of TNBC patients. Taken together, our data demonstrate a novel role for Smad3 in cancer transformation and cancer progression through TMEPAI and further suggest that selective targeting of TGF-β-Smad3-TMEPAI axis may be beneficial in triple negative breast cancer therapy and prevention.

## INTRODUCTION

Triple-negative breast cancers (TNBC), which constitute 15% to 20% of all breast cancers, are generally very aggressive with high recurrence rates compared to hormone-receptor-positive and/or HER2-positive breast cancers. Transforming growth factor-beta (TGF-β) is an important regulator that inhibits cell cycle progression of lobular and ductal epithelial cells to induce differentiation during normal mammary gland development and induces apoptosis in epithelial cells following cessation of lactation in the adult gland [1]. TGF-β is also involved in the development and progression of breast tumors through its secretion by the cancer cells [2, 3]. The role of TGF-β in the regulation of tumorigenesis is clearly reflected in the loss of sensitivity to TGF-β-induced growth inhibition causing cancer progression [4, 5]. The well documented dependency of triple negative breast cancers on TGF-β signaling activity for their growth and metastasis [6-9] provides a strong basis for developing novel therapeutic targets. TGF-β is the prototypic member of the TGF-β superfamily of dimeric growth factors that comprises of activins and bone morphogenic proteins [10, 11] and mediates a variety of biological effects on numerous cell types. TGF-β ligand and type I and type II receptors (TβR-I and TβR-II) on the cell surface form a complex, which is required to initiate the TGF-β signaling pathway. Binding of TGF-β ligand with type II receptor transactivates the type I receptor, which phosphorylates Smad2 and Smad3 proteins (regulatory members of Smad family proteins; R-Smads). These receptor activated R-Smads then form a ternary complex with a common Smad (Co-Smad), Smad4 and translocate into the nucleus to regulate transcription of several target genes through physical interaction and functional cooperation with other transcription factors and coactivators [11, 12]. Thus, TGF-β mediated R-Smad activation correlates with reduced proliferation of normal cells.

In the context of the role played by Smad mediated TGF-β signaling in several biological functions, it has been noticed that dysfunction of TGF-β signaling pathway has been linked to diverse set of developmental disorders and diseases, including cancer, fibrosis and autoimmune diseases [13]. TGF-β mediated Smad signaling pathway is involved both in growth inhibition of early stage cancers, a tumor suppressive effect of TGF-β, and causes epithelial-mesenchymal transition (EMT), a tumor promoting effect of TGF-β in advanced cancers [14-18]. Although targeting TGF-β signaling has a strong potential to treat cancers, hijacking crucial biological functions by antagonizing TGF-β signaling pathway carry the risk of disturbing the tumor suppressive homeostatic control of TGF-β in normal tissues and early cancers. Resistance to TGF-β-induced growth inhibition in cancer cells may be caused by several mechanisms, including i) inactivating mutations in components of the TGF-β /SMAD signaling pathway, ii) reduced expression of TGF-β/SMAD signaling components, and iii) altered expression of inhibitory molecules of the TGF-β/SMAD pathway. While the inactivating mutations in Smad2 and Smad3 have not been reported in breast cancer, this has been reported in Smad4 [19, 20]. Limited or no expression of Smad4, TβR-I or TβR-II due to mutation or aberrant expression, which may contribute to lack of TGF-β responsiveness are rarely observed in breast cancers. However, it was reported that functional differences exist between structurally similar Smad2 and Smad3 with respect to their target genes [21, 22], selective R-Smad deficiency leading to defects in embryonic development [23-25], Smad3 but not Smad2 dependent promotion of EMT in keratinocytes [26] and metastasis in breast cancer cells [27]. Earlier studies from our laboratory showed that breast cancer cells escape TGF-β mediated growth inhibition by overexpressing transmembrane prostate androgen induced (TMEPAI/PMEPA1), a direct target gene of Smad-dependent TGF-β signaling [14, 15, 28].

In the present study, we wished to address the underlying mechanisms for the pro-oncogenic behavior of TGF-β. Based on the results published, we hypothesized that aberrant functional expression of Smad2 and Smad3 in breast cancer contributes to the pro-oncogenic activities of TGF-β. Towards this, we assessed the relative contributions of Smad2 and Smad3. We used individual knockdown of R-Smads instead of overexpression to accurately reflect the functional state of the endogenous molecules [29, 30]. Our results identified that levels of Smad2 are downregulated in many triple negative breast cancer cells so that Smad3 can positively influence TMEPAI expression, which converts growth inhibitory Smad signaling into growth promoting non-Smad signaling that also promotes cell invasiveness and metastasis.

## RESULTS

### Altered expression levels of Smad2 and Smad3 in TNBC cell lines

**Figure 1:**
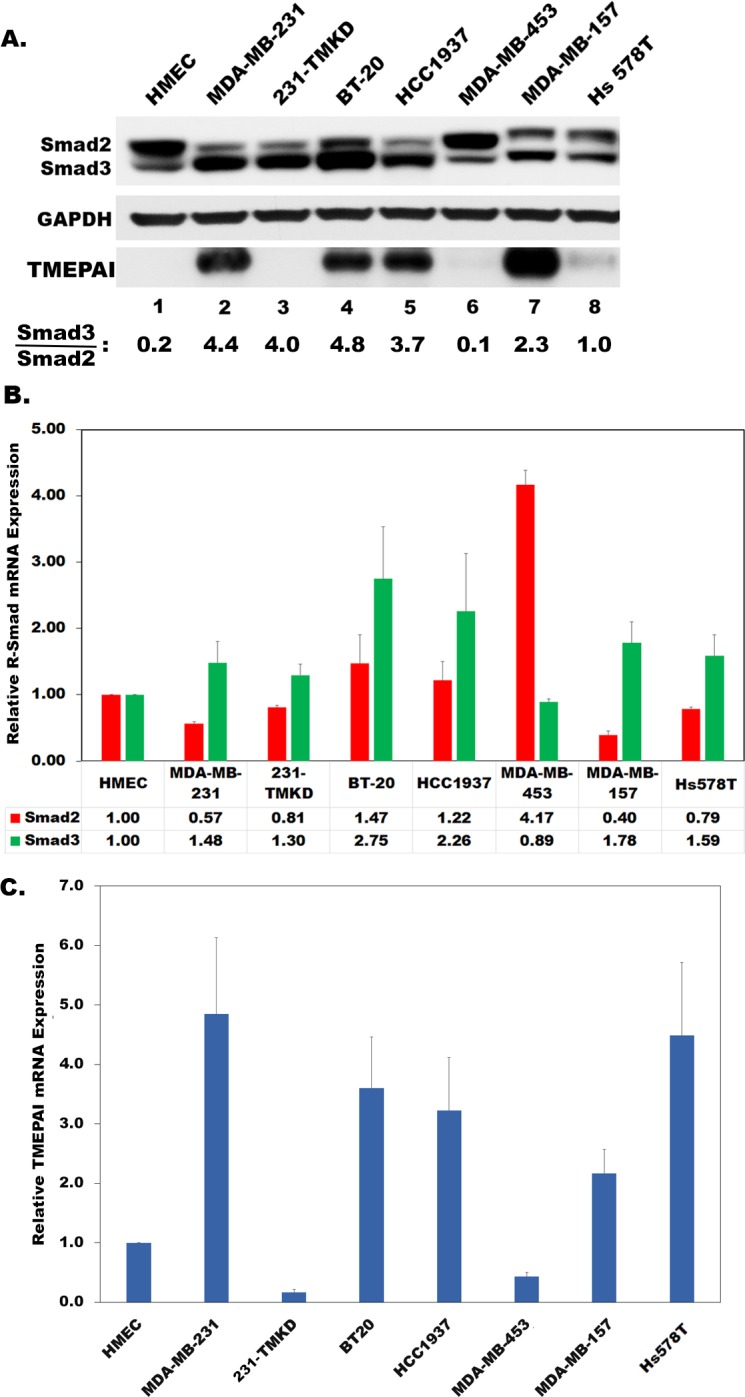
Relative expression of Smad2, Smad3 and TMEPAI/PMEPA1 proteins **A.,** and Smad2 and Smad3 mRNA **B.**; and TMEPAI mRNA **C.** in human normal mammary epithelial cells (HMEC) and various triple negative breast cancer cell lines (MDA-MB-231, BT20, HCC1937, MDA-MB-157, Hs578T) along with TMEPAI knockdown MDA-MB-231 cells (231-TMKD/TMKD).

Earlier, it was reported that reduction or loss of Smad2 together with Smad3 resulted in the promotion of skin carcinogenesis [26]. This prompted us to determine whether altered expression of Smad2 and Smad3 contributes to breast carcinogenesis. To address this, we selected various TNBC cell lines to represent all subtypes of TNBC [36] including unclassified (BT20) to Basal like (BL1; HCC1937), mesenchymal-like (MSL; MDA-MB-157, MDA-MB-231, HS578T) and a luminal androgen receptor (LAR; MDA-MB-453) subtypes as a model system along with human normal mammary epithelial cells (HMEC). Smad2 protein levels were found to be higher than Smad3 giving a low ratio of Smad3/Smad2 (0.2; Figure 1A) by immunoblot analysis in HMEC. However, this ratio is increased in most triple negative breast cancer cells (Smad3/Smad2 > 1.0; Figure 1A) except for MDA-MB-453 cell line (Smad3/Smad2 = 0.1; Figure 1A). Along with Smad2 and Smad3, TMEPAI level was also analyzed. While HMEC, and MDA-MB-453 did not register detectable levels, other cell lines generally showed high levels of TMEPAI. As expected, MDA-MB-231 cells showed no TMEPAI protein upon knockdown of TMEPAI using lentiviral vectors expressing shRNA without any change at Smad2 and Smad3 level. Measurement of relative mRNA by qPCR indicated an increased Smad3 mRNA levels in TNBC relative to normal HMEC except for MDA-MB-453 cells (Figure 1B). In contrast, Smad2 mRNA levels in TNBC are lower than normal HMEC except for MDA-MB-453, where they are elevated by 4 fold (Figure 1B). Notably, high Smad3/Smad2 ratio in TNBC correlated with high expression of TGF-β responsive gene, TMEPAI/PMEPA1 in these cells (Figure 1A and 1C). Since TMEPAI knockdown (TMKD) had little or no effect on the Smad3/Smad2 ratio, it suggests that TMEPAI may be a downstream effector of R-Smads (Figure 1). These observations, raise the possibility that Smad3 and Smad2 expression levels may be altered during tumor progression resulting in high TMEPAI expression in order to subvert growth suppressive TGF-β signaling into growth promotion [15].

### Effect of Smad2 and Smad3 on TGF-β mediated TMEPAI expression and breast cancer cell growth

**Figure 2:**
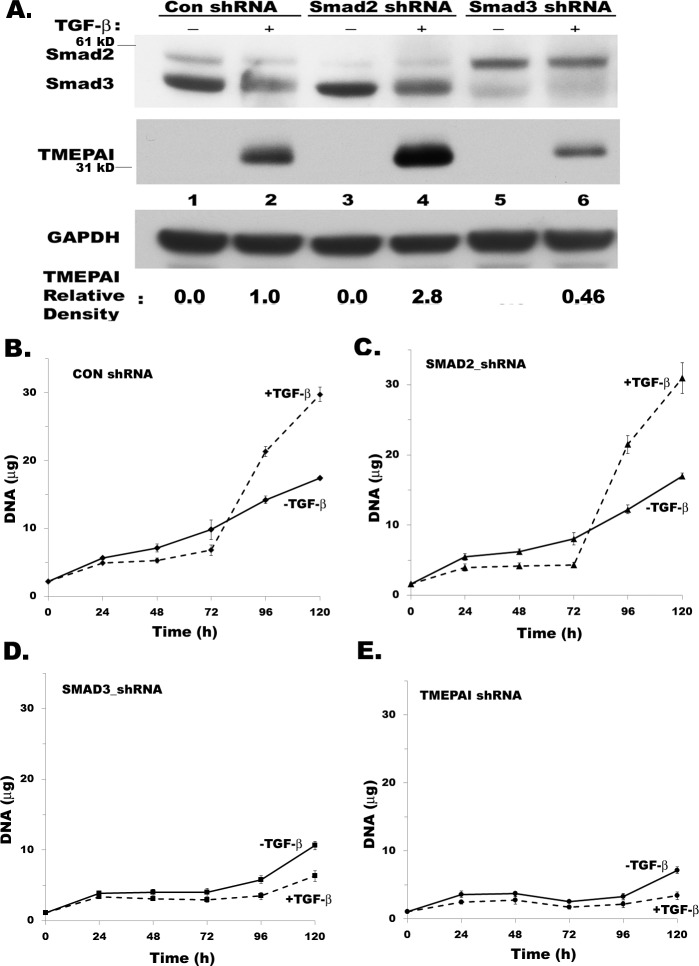
Effect of R-Smad knockdown and TGF-β on MDA-MB-231 breast cancer cell growth **A.** Relative levels of Smad2, Smad3, TMEPAI and GAPDH in MDA-MB-231 cells expressing control shRNA, Smad2 shRNA and Smad3 shRNA. **B.-E.** Growth curves of MDA-MB-231 cells expressing control shRNA (B), Smad2 shRNA (C), Smad3 shRNA (D) and TMEPAI shRNA (E) in the absence or presence of TGF-β (2 ng/ml).

We previously reported that TGF-β signaling has a critical role in promoting breast cancer cell growth and motility and silencing TMEPAI in TNBC cells inhibited tumor growth and metastasis in vivo [14, 15]. Since TMEPAI negatively regulates TGF-β mediated canonical Smad signaling in TNBC cells [15, 28], we examined the direct role of R-Smads on the TGF-β dependent growth of breast cancer cells by selective knockdown of individual R-Smads. Specifically, Smad3 deficiency, which lowered TMEPAI expression (Figure 2A compare lanes 2 and 6), dramatically inhibited the growth of MDA-MB-231 cells (Figure 2D) similar to TMEPAI knockdown (Figure 2E). In contrast, Smad2 deficient cells behaved like control cells (see Figure 2C and 2B) with increased active Smad3 and higher expression of TMEPAI (Figure 2A, compare lanes 2 and 4) suggesting that TMEPAI expression may be positively regulated by Smad3 and negatively by Smad2.

### Alterations in Smad2 and Smad3 levels influence TMEPAI/PMEPA1 transcription

**Figure 3:**
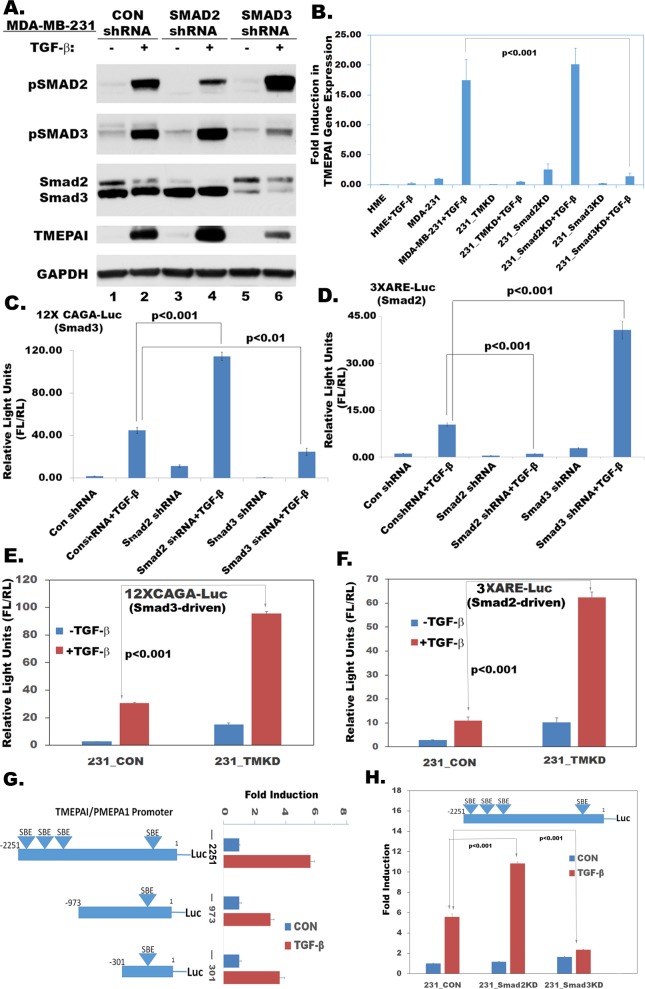
Effect of gene knockdown on R-Smad mediated transcriptional activity **A.** Relative expression of phospho Smad2/3, Total Smad2/3 and TMEPAI in MDA-MB-231 cells expressing Con shRNA, Smad2 shRNA and Smad3 shRNA in the absence or presence of TGF-β (2 ng/ml), **B.** Relative levels of TMEPAI mRNAs in human mammary epithelial cells (HME) and MDA-MB-231 cells that are expressing Con shRNA, TMEPAI shRNA (TMKD), Smad2 shRNA (Smad2KD) or Smad3 shRNA (Smad3KD) in the absence or presence of TGF-β (2 ng/ml), **C.** 12XCAGA-Luc reporter activity in MDA-MB-231 cells expressing Con ShRNA, Smad2 shRNA, Smad3 shRNA both in the absence or presence of TGF-β (2 ng/ml). **D.** 3XARE-Luc reporter activity in MDA-MB-231 cells expressing Con ShRNA, Smad2 shRNA, Smad3 shRNA both in the absence or presence of TGF-β (2 ng/ml). **E.** 12xCAGA-Luc reporter activity in MDA-MB-231 cells expressing Con ShRNA (231_CON) and TMEPAI shRNA (231_TMKD) in the absence or presence of TGF-β (2 ng/ml). **F.** 3XARE-Luc reporter activity in MDA-MB-231 cells expressing Con ShRNA (231_CON) and TMEPAI shRNA (231_TMKD) in the absence or presence of TGF-β (2 ng/ml). **G.** Schematic representation of three different size TMEPAI promoters tagged with luciferase reporter (−2251-Luc, −973-Luc, −301-Luc) and corresponding luciferase reporter activities in MDA-MB-231 cells in the absence or presence of TGF-β (2 ng/ml). **H.** TMEPAI promoter activity in MDA-MB-231 cells expressing con shRNA, Smad2 shRNA, and Smad3 shRNA in the absence or presence of TGF-β (2 ng/ml) using 2251 bp TMEPAI promoter (−2251-Luc).

When Smad2 and Smad3 deficiencies were monitored by Smad2/Smad4 responsive 3XARE-luciferase reporter and Smad3/Smad4 responsive 12XCAGA-luciferase reporter in wildtype and R-Smad knockdown MDA-MB-231 cells, Smad3 deficiency resulted in significant decrease of TGF-β-induced 12XCAGA-luc reporter activity (Figure 3C) and increase of 3XARE-luc reporter activity (Figure 3D) indicating availability of more active Smad2 in Smad3 deficient cells (Figure 3A). Correspondingly, Smad2 deficiency resulted in reduced 3XARE-luc reporter activity (Figure 3D) and increased 12XCAGA-luc reporter activity (Figure 3C) indicating that more activated Smad3 may be present in Smad2 deficient cells (Figure 3A). Interestingly, TMEPAI deficiency enhanced both 12XCAGA-Luc (Figure 3E) and 3XARE-Luc activities (3F). However, TMEPAI deficiency blocked cell growth like Smad3 deficiency. This is consistent with the notion that both Smad3 and TMEPAI deficiencies are functionally similar and lead to enhanced Smad2 transcriptional activity (3XARE-Luc activity; Figure 3D and 3F), which appears to be growth inhibitory.

To determine how TMEPAI/PMEPA1 promoter was regulated by TGF-β signaling, different lengths of TMEPAI promoter sequence, spanning from −2251, −971, or −301 to +119 bp relative to the transcription initiation site, were fused with a basic luciferase reporter that did not contain any promoter sequence or TATA box. There were 4 Smad binding elements (SBE) in 2.25 kb length promoter at positions −118, −1393, −1690 and −2014 from the transcription initiation site. When MDA-MB-231 cells were transfected and tested with above three promoter-luciferase fusion reporter plasmids, the longest promoter fragment gave higher TGF-β stimulated luciferase activity (Figure 3G) and was used for subsequent studies. To identify the role of R-Smad proteins in regulating TMEPAI expression, we tested the effect of deletion of individual R-Smads on the TMEPAI promoter in MDA-MB-231 cells. While Smad2 deficiency significantly stimulated TGF-β induced TMEPAI promoter activity, Smad3 deficiency considerably inhibited it (Figure 3H) suggesting that TMEPAI expression is dependent on Smad3 transcriptional activity. Overall, these results indicate that TGF-β-induced TMEPAI promoter activity is positively regulated by SMAD3 and negatively by SMAD2.

### Smad3-TMEPAI axis is required for growth and invasive behavior of TNBC

**Figure 4:**
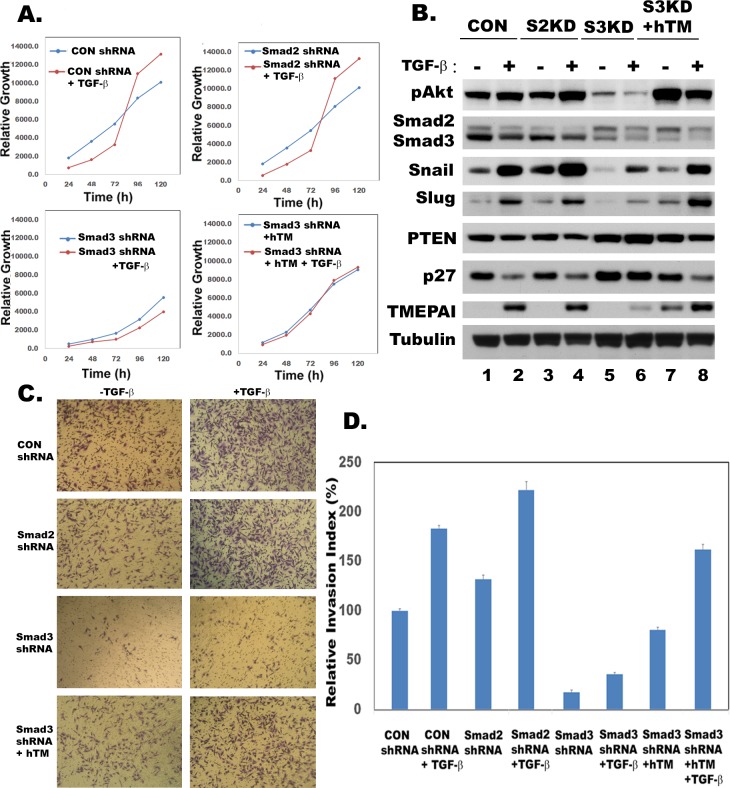
TMEPAI reverses Smad3 gene knockdown effects **A.** Growth curves of MDA-MB-231 cells expressing control shRNA (CON shRNA), Smad2 shRNA, Smad3 shRNA or Smad3shRNA along with human TMEPAI cDNA (Smad3 shRNA+hTM) in the absence or presence of TGF-β (2 ng/ml). **B.** Relative expression of phosphorylated Akt (pAkt), Smad2, Smad3, Snail, Slug, PTEN, p27 and TMEPAI in MDA-MB-231 cells expressing Con shRNA (CON), Smad2 shRNA (S2KD), Smad3 shRNA (S3KD) and Smad3 shRNA along with human TMEPAI cDNA (S3KD+hTM) in the absence or presence of TGF-β (2 ng/ml). Phase contrast images of toluidine blue stained transit well invasion assay (C) and relative invasion index (D) of MDA-MB-231 cells expressing control shRNA (CON shRNA), Smad2 shRNA, Smad3 shRNA and Smad3shRNA along with human TMEPAI cDNA in the absence or presence of TGF-β (2 ng/ml).

**Figure 5:**
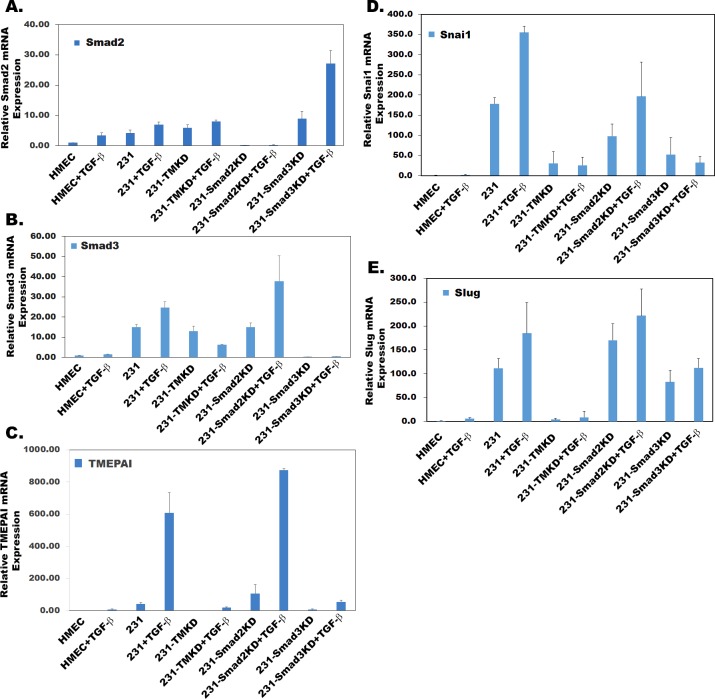
Effect of gene knockdown on metastasis promoting genes Relative mRNA expression of Smad2 **A.**, Smad3 **B.**, TMEPAI **C.**, Snai1 **D.**, Slug **E.** in MDA-MB-231 (231) cells that are expressing control, TMEPAI (TMKD), Smad2 (Smad2KD) and Smad3 (Smad3KD) shRNAs in the absence or presence of TGF-β (2 ng/ml) relative to normal human mammary epithelial cells (HMEC).

Since both Smad3 and TMEPAI knockdown had identical effect on breast cancer cell growth (Figure 2D and 2E), we tested whether TMEPAI expression driven by Smad3 is responsible for subversion of growth and migration suppressive effects of TGF-β in breast cancer cells. This was addressed by exogenously overexpressing human TMEPAI (a full length 287 amino acid isoform) in Smad3 knockdown cells that were growth inhibited. Indeed, TMEPAI expression enhanced the growth of Smad3 knockdown cells (Figure 4A, compare bottom left and right panels) both in the absence and presence of TGF-β. Lack of TGF-β response is due to constitutive expression of TMEPAI that is not regulated by TGF-β. Since we showed earlier that growth subversion due to TMEPAI occurs through PTEN [15]. Indeed Smad3 knockdown not only abrogated TGF-β induced TMEPAI levels, it also increased PTEN levels with reduced Akt activation (pAkt) under both basal conditions as well as in presence of TGF-β (Figure 4B), which was reversed by exogenous expression of TMEPAI(Figure 4B). Similarly, endogenous cell cycle inhibitor, p27, whose levels are low in control and Smad2 deficient cells, is elevated in Smad3 deficient cells (Figure 4B) and exogenous expression of TMEPAI in Smad3 deficient cells caused reduction in p27 and PTEN (Figure 4B), which promoted cell growth (Figure 4A). Since activated PI3K/Akt signaling not only plays an important role in growth but also in the induction of EMT by stabilizing Snail [37], we analyzed Snail and Slug proteins that are involved in regulating invasiveness of tumor cells. Earlier, we have reported that TMEPAI knockdown reduced snail levels and inhibited cell migration, invasion and metastasis both in vitro and in vivo [15]. Because TMEPAI is a Smad dependent TGF-β target gene [15, 28] and Smad3 but not Smad2 deficiency reduced TMEPAI levels (Figure 3A), we tested the effect of R-Smad deficiency on cell migration and invasion of breast cancer cells by using Boyden chamber coated with matrigel. As shown in Figure 4C, while control and Smad2 deficient MDA-MB-231 cells invaded through matrigel, which is further enhanced in presence of TGF-β (Figure 4C and 4D), smad3 deficient cells had reduced ability to invade through matrigel both in the absence and presence of TGF-β (Figure 4C and 4D). Moreover, Snail and slug, two important proteins involved in EMT phenomena showed significant reduction in Smad3 deficient cells compared to control and Smad2 deficient cells (Figure 4B). As expected, exogenous expression of TMEPAI reversed Snail and Slug reductions seen in Smad3 deficient cells (Figure 4B) and significantly enhanced their ability to invade (Figure 4C and 4D) through matrigel. When the relative expression levels of Smad2, Smad3, TMEPAI, Snai1 and Slug mRNAs using q-PCR in R-Smad deficient cells were determined, the reciprocal increase in Smad3 mRNA in Smad2 deficient cells and vice versa (Figure 5A and 5B) was much more pronounced in the presence of TGF-β. Similarly, decreased TMEPAI mRNA levels in Smad3 deficient cells and increased TMEPAI mRNA levels in Smad2 deficient cells are also more dramatic in presence of exogenous TGF-β (Figure 5C). In line with their reduced migration through matrigel, significant reduction in both Snai1 and Slug mRNAs was seen in Smad3 deficient cells (Figure 5D and 5E). Although slight reduction in Snail mRNA was seen in Smad2 deficient cells (Figure 5D), it did not affect Snail protein levels (Figure 4B).

**Figure 6:**
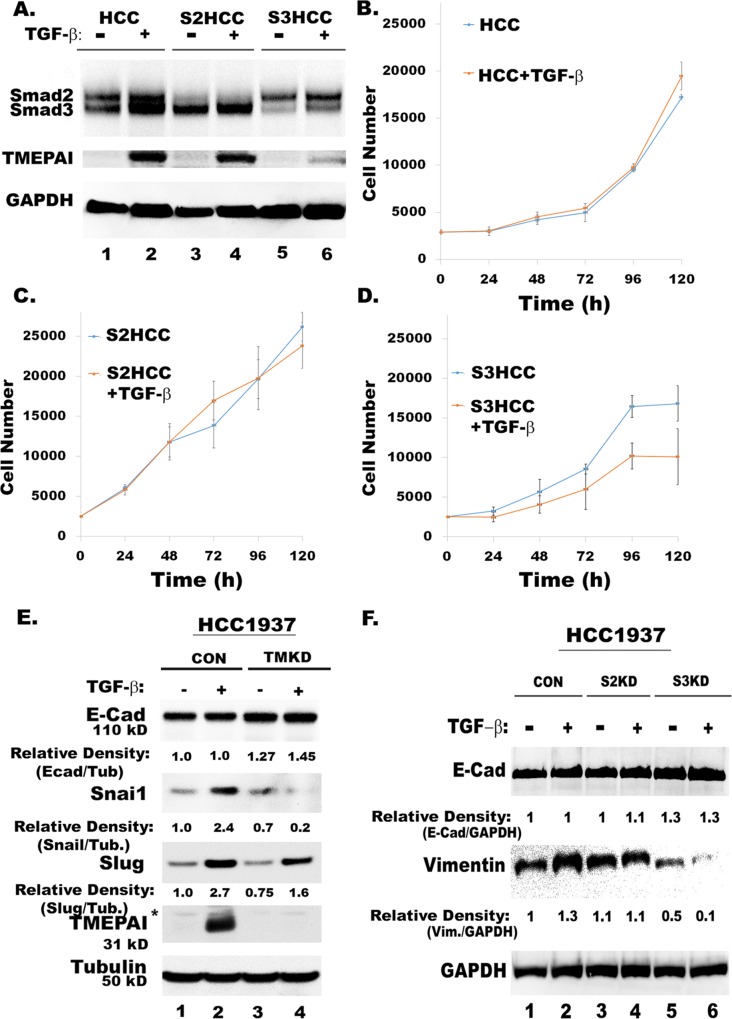
Knockdown of TMEPAI or Smad3 promotes epithelial markers and reduces mesenchymal and metastatic markers in HCC1937 cells **A.** Relative expression of Smad2, Smad3 and TMEPAI in Control (HCC), Smad2 knockdown (S2KD) and Smad3 knockdown (S3KD) HCC1937 TNBC cells. **B.** Growth of control HCC1937 (HCC) in the presence and absence of TGF-β. **C.** Growth of Smad2 knockdown HCC1937 (S2KD) in the presence and absence of TGF-β. **D.** Growth of Smad3 knockdown HCC1937 (S3KD) in the presence and absence of TGF-β. **E.** Relative expressions of E-Cad, Snail and Slug in control (CON) and TMEPAI knockdown (TMKD) HCC1937 cells. **F.** Relative expressions of E-Cad and Vimentin in control (CON), Smad2 knockdown (S2KD) and Smad3 knockdown (S3KD) HCC1937 cells.

To further confirm the role of Smd3 and TMEPAI/PMEPA1 in promoting metastasis, we addressed their effect in regulating direct EMT genes along with EMT associated Snail and Slug genes in response to canonical TGF-β stimulation. Since MDA-MB-231 cells do not express the epithelial marker, E-Cadherin, we used another TNBC cell line, HCC1937 that expresses E-Cadherin. While TGF-β has no effect on the growth of both wild type and Smad2 knockdown (Smad2KD) cells, it had inhibited the growth of Smad3 knockdown (Smad3KD) cells (Figure 6B-D). Just like in MDA-MB-231cells (Figure 2A), Smad3 knockdown in HCC1937 cells (Smad3KD) results in low expression of TMEPAI (Figure 6A). Unlike highly motile MDA-MB-231 cells, HCT1937 cells express very low levels of Vimentin (not shown) but express E-Cadherin (Figure 6E). TMEPAI/PMEPA1 knockdown relatively increases E-Cadherin levels and reduces both Snail and Slug protein expressions (Figure 6E). Similarly, Smad3 knockdown results in higher expression of E-Cadherin and low expression of vimentin (Figure 6F), which supports the idea that SMAD3/TMEPAI signaling axis may overall regulate transcriptional control of EMT program.

### Low Expression of Smad2 and high expression of TMEPAI are associated with decreased survival

**Figure 7:**
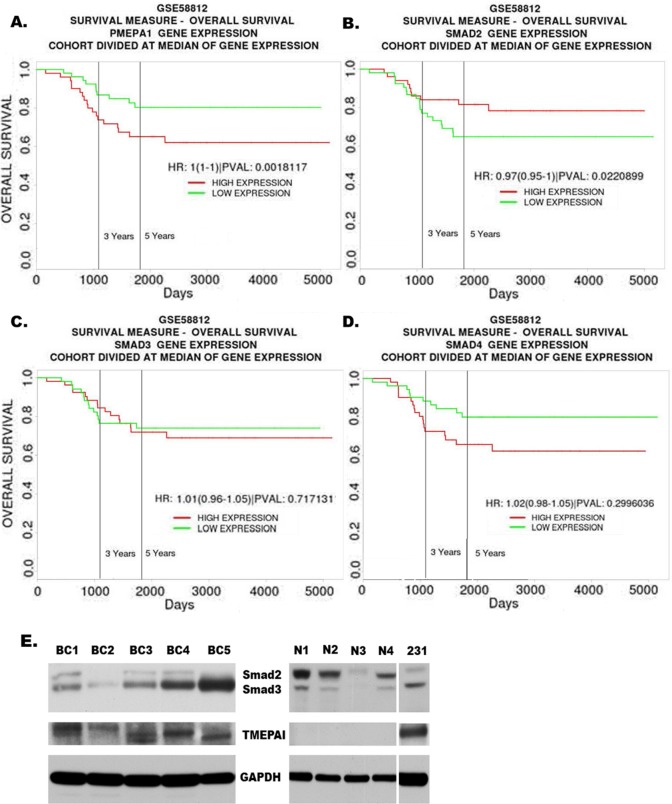
High Expression of TMEPAI/PMEPA1 and low expression of Smad2 are associated with poor prognosis of TNBC patients Kaplan Meier curves for overall survival of triple negative breast cancer patients in relation to expression of TMEPAI/PMEPA1 (A), Smad2 (B), Smad3 (C) or Smad4 (D) mRNA using a publicly accessible database (GSE58812). E. Western blots of TMEPAI and total Smad2/3 proteins in primary tumors from human breast cancer patients (BC1- BC5) and corresponding normal/benign (N1-N4) samples relative to MDA-MB-231 cells (231).

Based on the cellular functions associated with differential expression of TMEPAI/PMEPA1, Smad2 and Smad3, we reasoned that TNBC patients may likely have different outcomes due to variations in their expression levels. We examined human cancer databases to address this question. We used the PROGgene V2 online tool [35, 38] to compare the prognostic value of TMEPAI/PMEPA1, Smad2, Smad3 and Smad4 gene expressions in triple negative breast tumor datasets [34]. The triple negative breast cancer patients with higher TMEPAI/PMEPA1 and lower Smad2 mRNA expression showed decreased survival (Figure 7A and 7B). Higher expression of Smad3 and Smad4 are also associated with decreased survival, but it was not statistically significant (Figure 7C and 7D). We also carried out the analysis of TMEPAI, Smad2 and Smad3 proteins in primary tumor tissues of TNBC. The tumor lysates of five TNBC patients (BC1-BC5) and four normal/benign tissues (N1-N4) showed similar differences in Smad2 and Smad3 protein expression levels in tumors vs normal/benign tissues (Figure 7E) as was noted in established cell lines (Figure 1). Overall, our findings show clinical significance for high TMEPAI and low Smad2 in triple negative breast cancers and confirm the need to further understand the role and the regulation of TMEPAI.

## DISCUSSION

Triple negative breast cancers (TNBC) depend on TGF-β mediated Smad signaling activity for their growth and metastasis. Since TGF-β suppresses the growth of normal epithelial cells and early stage cancer cells, it is likely that either defects or alterations in Smad signaling pathway may result in breast carcinogenesis of triple negative type. Although earlier inferences regarding the dual role of TGF-β in carcinogenesis have been obtained using different in vitro and in vivo models, a comparative analysis of Smad2 and Smad3 proteins allowed us to determine how such a functional switch truly occurs in breast cancer. Based on our results, for the first time, we can state that tumor suppressive TGF-β signaling pathway is dependent on Smad2 mediated activities and pro-oncogenic TGF-β signaling may dependent on Smad3 mediated activities. Further, we have also provided supportive data to show that Smad3 dependent expression of TMEPAI/PMEPA1 contributes to the growth of cancer cells and invasion.

While the mechanisms through which breast cancer cells escape TGF-β mediated growth suppression are being pursued, we observed a consistent reduction in Smad2 protein in several human triple negative breast cancer cells relative to normal cells. It is likely that the reduction of Smad2 protein could result from effect at the transcriptional and /or posttranslational level. Similar alterations at the transcript level were also noticed in TNBC cells compared to normal cells. Smad2 reduction elevates the Smad3 to Smad2 ratio even with or without Smad3 increase, which seemed to favor increased expression of TMEPAI both at transcript and protein levels. The published scientific literature on TMEPAI/PMEPA1 shows that there are multiple variants or isoforms [39]. Our investigations addressing the functions of TMEPAI/PMEPA1 have utilized only a cDNA clone encoding 287 amino acid variant. Previously, relative mRNA expressions of TGF-β receptors and R-Smads were compared in various triple negative breast cancer cell lines, but the data failed to correlate TGF-β resistance to altered Smad or TGF-β receptor mRNA expression levels [40, 41] because of breast cancer heterogeneity that included all types of breast cancers. Studies that have shown different functional roles of Smad2 and Smad3 in cancer cells [27, 42, 43] never compared the expression of Smad2 and Smad3 at protein level in cancer cells versus normal cells. Since Smad2 undergoes LOH in some cancers [44], it is considered as a tumor suppressor, which was supported by the observation that its expression at mRNA level was lower in invasive breast cancers compared to normal breast tissues [45]. Our studies are the first to report that cells may escape TGF-β mediated growth suppression in triple negative breast cancer by altering endogenous Smad2 and Smad3 protein levels. Since alteration of protein ratios depend on their relative expressions and turnovers, it is unlikely that relative affinities of Smad2 and Smad3 would dictate their altered ratios. Here we would like to point out that IHC technology may not be suitable to quantitatively distinguish these R-Smads by using either a single antibody for both or specific antibodies to each.

In an attempt to understand the role of the TGF-β - Smad - TMEPAI axis in promoting breast carcinogenesis, we studied relative contributions of Smad2 and Smad3 to the growth of breast cancer cells by altering endogenous Smad2 and Smad3 protein levels using shRNA as this approach mimics altered Smad3 to Smad2 ratios observed in cancer cells. In our opinion, this is a better strategy than gene overexpression or complete gene knockout approaches and avoids over-gain or complete loss of gene function. Unlike in mice, where Smad2 deficiency is embryonic lethal [46] and Smad3 deficient mice are viable and fertile [47], Smad3-deficiency in MDA-MB-231 triple negative breast cancer cells retarded their growth similar to TMEPAI knockdown [14, 15] and Smad2 deficient cells behaved like wildtype cells (Figure 2). The common denominators between Smad3 and TMEPAI deficient cells are high Smad2 transcriptional activity and low TMEPAI expression, which resulted in reduced cell growth In contrast, high Smad3 transcriptional activity combined with high TMEPAI expression promoted breast cancer cell proliferation suggesting that Smad3 may function through TMEPAI. Indeed, Smad3 deficiency resulted in low expression of TMEPAI both at protein and mRNA levels. In contrast, Smad2 deficient cells expressed more TMEPAI than even control cells suggesting that Smad2 may negatively regulate TMEPAI expression. Moreover, TMEPAI promoter activity is high in Smad2 deficiency and low with Smad3 deficiency. In TMEPAI knockdown cells, even though both Smad2 and Smad3 activities are enriched, the growth of breast cancer cells is inhibited (Figure 2E) suggesting that Smad2 transcriptional activity is dominant and growth inhibitory over Smad3 in the absence of TMEPAI. On the other hand, Smad3 promotes growth by stimulating TMEPAI expression, which reduces PTEN, through PI3K/Akt signaling [15] in cells with a reduced or loss of Smad2,. Indeed, above observations are in agreement with the findings of Petersen et al. [27], who reported opposing roles of Smad2 and Smad3, where Smad3 drives breast cancer bone metastasis.

A significant finding from our studies is that Smad3 is needed for TMEPAI expression. This is further supported by exogenous TMEPAI overexpression in cells that rescued the growth and motility deficiency caused by Smad3 reduction. These results suggest that the driving mechanism for growth and metastasis of triple negative breast cancers is Smad3 driven TMEPAI expression. Previously, we showed a pro-metastatic role of TMEPAI in breast cancer lung metastasis using MDA-MB-231 cells [15]. Since PTEN protein levels are regulated by TMEPAI, the TGF-β –SMAD3-TMEPAI-PTEN axis may be involved in regulating both growth and motility of breast cancer cells. PI3K/Akt pathway can activate Snai1 and slug expression that has been correlated with EMT and invasive tumor types through repression of E-cadherin and occludin expression mediated by Snai1/SMAD3/SMAD4 complex [48]. We showed that blocking the function of TMEPAI in MDA-MB-231 breast cancer cells strongly suppressed the formation of metastatic foci in lungs of mice [15], thus supporting our Smad3-TMEPAI-PTEN axis hypothesis in triple negative breast cancer progression. Our results, which suggested that Smad3 is critical for stimulation of tumor growth and metastasis, are in line with the observation that overexpression of a C-terminal truncated dominant negative mutant of Smad3, which inhibited TGF-β-induced migration of MCF10A cells [49].

**Figure 8:**
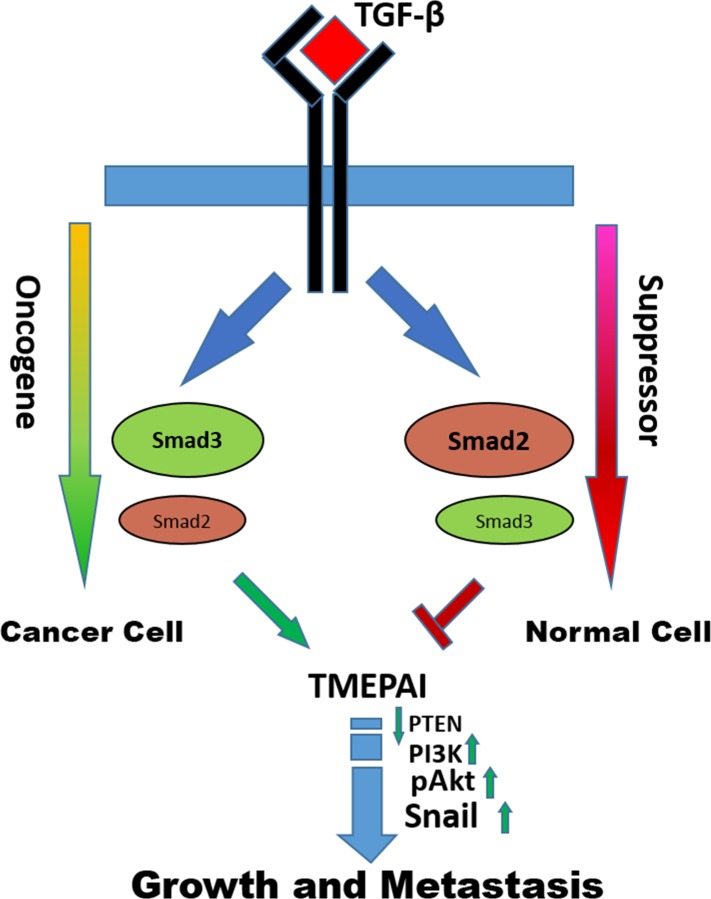
Model of the opposing effects of Smad2 and Smad3 signaling in cancer cells and normal cells The data demonstrate that the consequences of switch from Smad2 to Smad3 signaling pathway results in converting TGF-β from tumor suppressor to pro-oncogene as cells acquire increasing malignant properties through Smad3 mediated TMEPAI expression.

Based on the results presented in the manuscript, we have proposed a model for breast cancer progression (Figure 8). During breast tumorigenesis it seemed necessary to turn off TGF-β-mediated Smad2 responses while retaining the Smad3 responses for further progression of breast tumors of triple negative type. The mechanisms by which Smad2 loss and Smad3 gain may occur from genetic alterations that reduce or increase transcript levels and/or posttranslational modifications leading to altered protein turnover. The results presented here support the idea that development of several triple negative breast cancers may involve expansion of cell populations with altered Smad2 and Smad3 levels resulting in Smad3 dependent expression of TMEPAI, which provides a competitive advantage for cancer cells to grow and metastasize in presence of TGF-β. Identification of specific molecules like TMEPAI that are involved in blocking the TGF-β-mediated growth suppression will facilitate the development of novel therapeutics and may improve responses to currently available therapies. It may be that other cancer types such as non-small cell lung adenocarcinomas and gastric cancers could also benefit from this approach as well. In fact, meta-analysis of published microarray datasets [50, 51] revealed that increased TMEPAI and Smad3 expression and decreased Smad2 expression in lung and gastric cancers was significantly associated with poorer patient prognosis (Supplementary Figure 1 and Supplementary Figure 2). The data on Smad4 is the most interesting which has the opposite effect of Smad2 (Figure 7). Since both are located on chromosome 18 it is common when these tumor suppressors are lost to cause poor prognosis. While this genetic lesion might be rare in TNBC it opens the door to the transcriptional control mechanism in a Smad2/4 vs Smad3/4 control of TMEPAI/PMEPA1.

In summary, we identified a marked decrease in Smad2 protein levels to increase the Smad3 to Smad2 protein ratio in several human triple negative breast cancer cell lines. We also identified opposing roles for Smad2 and Smad3 in the TMEPAI/PMEPA1 expression of TNBC, which promotes their growth and motility. These findings provide a mechanistic basis for future therapies that target TGF-β-Smad3-TMEPAI signaling axis in effectively treating and/or preventing triple negative breast cancers.

## MATERIALS AND METHODS

Materials: TGF-β (R&D, MN), Antibodies to TMEPAI/PMEPA1 (Abnova, (mouse monoclonal) Taiwan and Proteintech, (rabbit polyclonal) CA), pSmad3 (Rockland, PA), H33258 (Bisbenzimide) and Tubulin (Sigma, MO), GAPDH (R&D, MN), pSmad2, Smad2/3 pAkt, Akt, PTEN, Snail, Slug, p27 and Actin (Cell Signaling, MA).

### Cell culture

MDA-MB-231, BT-20, HCC1937 breast cancer cells and HMEC were maintained and grown as previously described [14, 15, 31]. MDA-MB-453, MDA-MB-157, Hs578T were purchased in the year 2015 from ATCC and grown according to the ATCC recommended culture conditions. All cell lines were authenticated by genomic STR profile.

### Cell proliferation assay

Cell proliferation was measured by quantitation of total cell DNA with Hoechst 33258 as mentioned before [14, 15, 32] or by water-soluble tetrazolium salt (WST-8) assay using microplate reader according to the protocol of the Cell Counting Kit-8 (CCK8) assay kit (Med Chem Express, USA). Briefly, cells were first fixed with ice cold 80% ethanol and washed with PBS. Then cells were incubated with Hoechst 33258 at 1µg/ml in PBS for 20 min at 37oC. After this, cells were washed with PBS and read at 360 nm (excitation) and 465 nm (emission) in Tecan plate reader.

### Cell invasion assay

MDA-MB-231 cells bearing control shRNA, Smad2 shRNA, Smad3 shRNA and Smad3 shRNA along with TMEPAI overexpression treated without or with TGF-β and used for Cell invasion assay as mentioned before [31].

### Western blot analysis

Western blots were performed and analyzed as described before [14, 15]. Briefly, Proteins from different cell lysates were resolved by SDS–polyacrylamide gel electrophoresis (SDS–PAGE) using 10% or 4–12% Bolt gels with appropriate running buffer (MES or MOPS buffers; ThermoFisher Scientific, USA) and were electroblotted onto PVDF membranes (0.45µm) in Pierce G2 fast blotter (ThermoFisher Scientific, USA) using manufacturer's instructions. Western blotting was performed on the above membranes using appropriate primary and secondary antibodies. Chemiluminescent substrates (ThermoFisher Scientific, USA) or Advansta Western bright (Advansta, CA) were used to detect antigen-antibody complexes on the PVDF membrane.

### Gene knockdown

TMEPAI knockdown was achieved using lentiviral vectors as mentioned before [14, 15]. Knockdown of Smad2 and Smad3 was achieved by using retroviral vectors from Addgene [33].

### Quantitative real-time-PCR

Quantitative RT-PCR was performed as described before (Singha et al., 2010, 2014) using Smad2, Smad3 and 18S rRNA primers. The nucleotide sequences for PCR primers were Smad2, 5′-AATGGCAAGATGGACGACA-3′ (forward) and 5′-GGAGAAGCAGCTCGCCA-3′ (reverse); Smad3, 5′-GTAGCTCGTGGTGGCTGTG-3′ (forward), 5′-ACGTCAACACCAAGTGCAC-3′ (reverse); 18SrRNA, 5′-GAGAAACGGCTACCACATCC-3′ (forward) and 5′-ACCAGACTTGCCCTCCA-3′ (reverse).

### Luciferase assays

For Smad2/Smad4 driven transcriptional activity, cells were transfected with 3X-ARE-Luc vector (a gift from Dr. Joan Massague (Addgene plasmid # 14934; http://n2t.net/addgene:14934;RRID:Addgene_14934) along with FLAG-FAST-1 expression vector. For Smad3/Smad4 driven transcriptional activity cells were transfected with 12XCAGA-Luc vector (a gift from Dr. Susumu Itoh). For TMEPAI promoter driven transcriptional activity cells were transfected with vectors containing three different size fragments of human TMEPAI promoter in tandem with firefly luciferase gene along with a renilla luciferase expression vector to normalize for transfection efficiency. Cells were transfected with Lipofectamine 3000 reagent following manufacturer's instructions (ThermoFisher, CA). Dual luciferase Reporter assay system from Promega was used to measure luciferase activities in transfected cells following various treatments according to vendor's instructions (Promega, WI). All experiments were repeated at least three times.

### Bioinformatics

The dataset GSE58812 [34] that was used for molecular subtyping triple negative breast cancer patient samples was used to compare Smad2, Smad3, Smad4 and TMEPAI/PMEPA1 expressions and assessed overall survival using the PROGgeneV2 tool [35] through comparison of high and low expression groups using median gene expression value as a dividing point.

### Statistical analysis

Data were expressed as the mean + S.D. Statistical analysis was performed using one-way analysis of variance (ANOVA), followed by Tukey's test, using Graph Pad prism software. All values were considered statistically significant when p<0.05.

## SUPPLEMENTARY FIGURES


