# Supplementary material for: Increased Smad3 and reduced Smad2 levels mediate the functional switch of TGF-β from growth suppressor to growth and metastasis promoter through TMEPAI/PMEPA1 in triple negative breast cancer
Source: Genes Cancer. 2019;10(5-6):134–49. doi: 10.18632/genesandcancer.194 (PMC6872668; doi:10.18632/genesandcancer.194)
Supplement: Supplementary file 1 [file ganc-10-134-s001.pdf]

Increased Smad3 and reduced Smad2 levels mediate the functional switch of TGF-β from growth suppressor to growth and metastasis promoter through TMEPAI/PMEPA1 in triple negative breast cancer

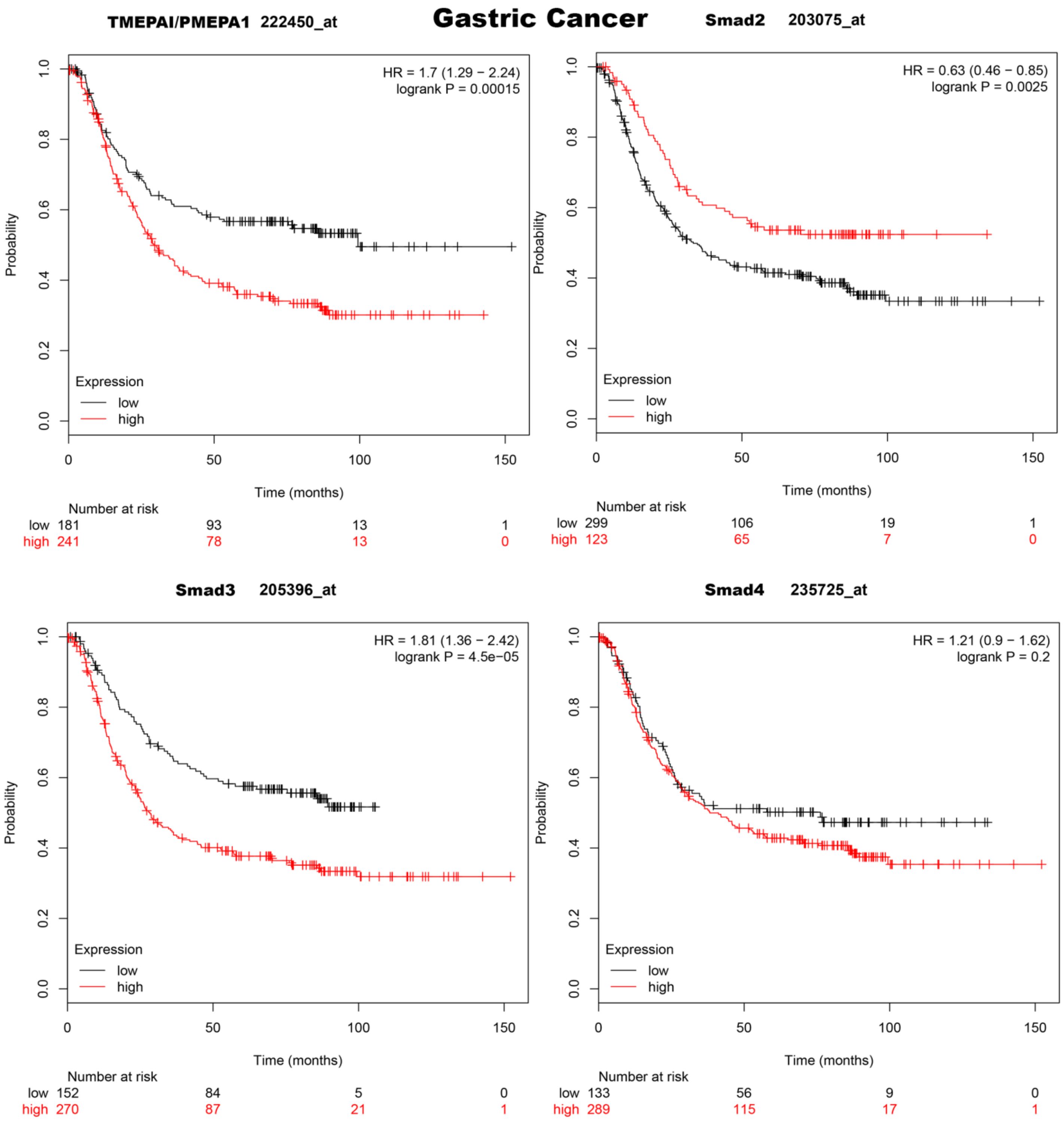

Supplementary Figure 1

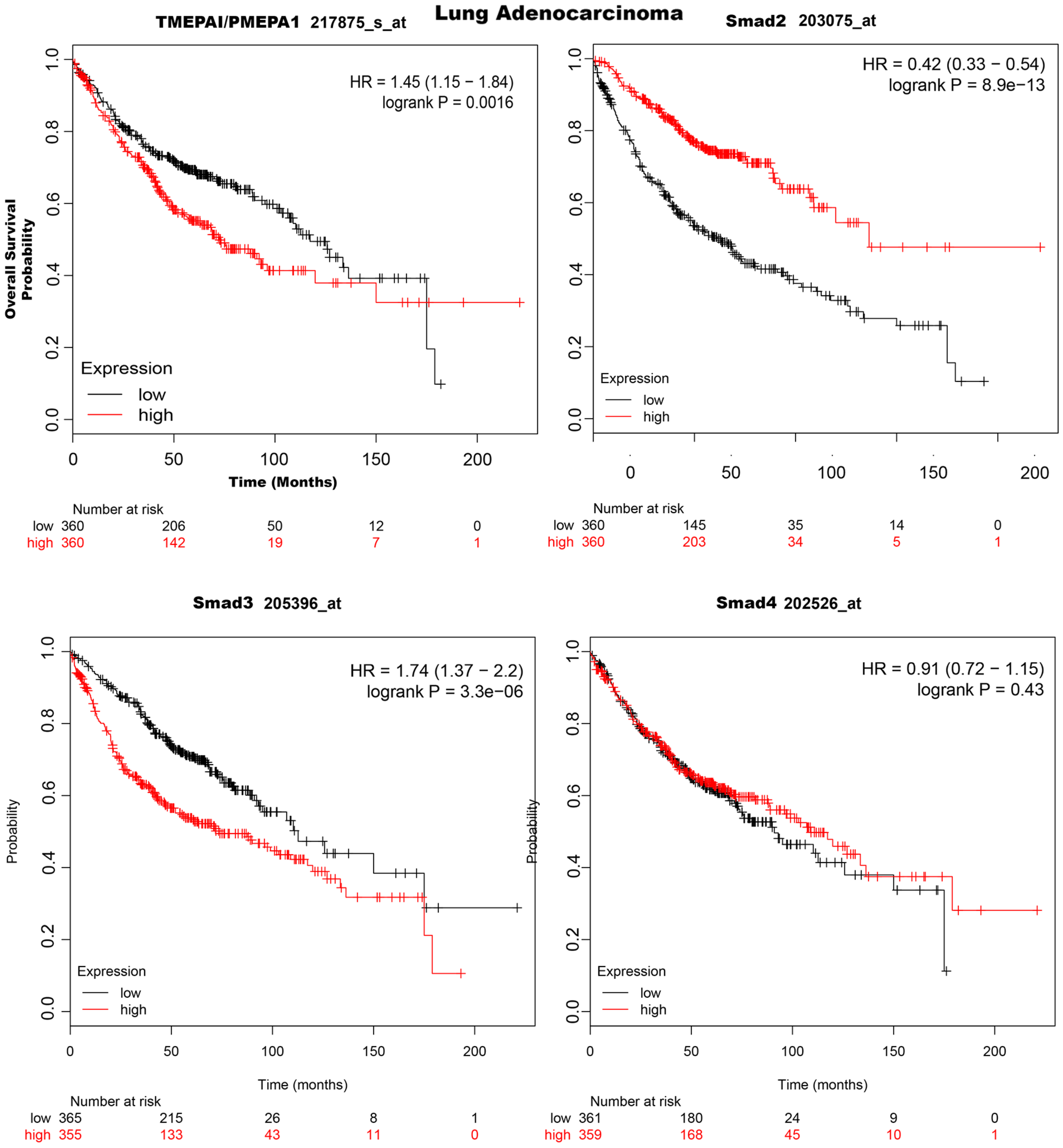

Supplementary Figure 2
